# Effects of brief exposure to campus environment on students' physiological and psychological health

**DOI:** 10.3389/fpubh.2023.1051864

**Published:** 2023-04-13

**Authors:** Wei Ning, Jiahui Yin, Qiang Chen, Xiaogang Sun

**Affiliations:** ^1^College of Forestry and Grassland Science, Jilin Agricultural University, Changchun, China; ^2^Zonbong Huize Landscape Environmental Construction Limited, Changchun, China

**Keywords:** psychological health, physiological health, university campus, transient recovery, nature exposure

## Abstract

Experiences in natural environments can enhance human wellbeing and promote the recovery of physiological and psychological health. During the COVID-19 pandemic, university students' activity off-campus was limited, thus, the campus environment was particularly important for the recovery of students' physiological and psychological health. Although the benefits of sustained natural exposure are obvious for people's physiological and psychological health, the effects of brief exposure on physiological and psychological are unclear. In the present study, four types of campus environments, including square space, dense forest space, sparse forest space, and waterfront space, were selected to explore the transient recovery effects of different types of campus environments. Sixty university students were recruited, measuring their systolic blood pressure, diastolic blood pressure, and heart rate as physical parameters to assess stress recovery. Respondents also reported scores about their personal feelings in questionnaires to evaluate their psychological states. Both physiological and psychological indicators responded to the brief natural exposure (5 min), and physiological and psychological health was restored. However, only the recovery amounts of psychological indicators were significantly different in waterfront space, dense forest space, and sparse forest space. These results indicate that being compare with other spaces, the brief exposure in the waterfront space was the most beneficial to students' psychological health recovery. This recovery was attributed to the great role played by the sense of escape, but after the brief exposure, the attraction and compatibility of the environment would hinder the psychological health recovery. In conclusion, according to tests on both physiological and psychological aspects, the waterfront environment on campus is the best choice for students' transient health recovery.

## 1. Introduction

Physiological stress can have a negative impact on human health, including the effects of acute or chronic stress and even inadequate recovery from stress (1, 2). The increase in stress correspondingly leads to physiological disorders and cardiovascular disease (3, 4). According to the survey, stress related to work or school, or university was mentioned most frequently (23.8%). Noise (6.1%), time pressure (4.9%), traveling to the present location (3.7%), and arguments or social conflicts (3.1%) were mentioned less frequently (5). Contemporary university students in East Asia have long become a high-pressure group in society. Their pressure comes from worries about the expansion of universities, employment difficulties, interpersonal relationships, and so on. They face increasing challenges to their physiological and psychological health. Since the World Health Organization declared the global pandemic of COVID-19, more anxiety may have been imposed on the stressed university students in addition to their stresses from academic lives on campus (6).

According to Wilson's Biophilia Hypothesis (1984), humans have a tendency to contact nature for the potential benefits of recovering physiological and psychological health (7, 8). It was demonstrated that watching forest landscapes, listening to natural sounds, and walking in actual forests may all lead to positive responses to emotional physiological states (9–12). Exposure to nature can reduce stress among teenagers, especially the stress of exams and the anxiety of campus life (13, 14). The greenness and diverse open spaces have positive effects on students' physiological and psychological rehabilitation (15, 16). Therefore, it is necessary to study the relationship between the campus environment and college students' physiological and psychological health.

Due to the characteristics of a cold climate, an aging population, and unbalanced economic development, cardiovascular diseases in northern China may be higher than those in other parts of China and have shown an obvious upward trend in recent years. In Jilin Province, the prevalence rate of hypertension among adults over 15 years old was 24.73% (17). In view of the asymptomatic nature of hypertension and the tendency of college students to suffer from cardiovascular diseases due to poor living habits and eating habits, attention should be paid to the cardiovascular health problems of college students in northeast China (18, 19). Some experiments have proved that blood pressure and heart rate can be used to measure physiological recovery (20–22). Ulrich studied the recovery response benefits of natural and urban landscapes by measuring heart rate (23). Staats and Hartig studied the resilience of natural and urban environments by measuring blood pressure (24). For mental health recovery, a systematic review indicated that forest therapy improved subjects' mental health (25). Some theories have been used as an important theoretical basis to explain the stress-relieving function of the natural environment. Ulrich's stress-relief theory suggests that stress causes a decrease in concentration, and he believes that the natural environment has a positive effect on people's emotions and physiology and has a significant effect on relieving mental stress (5). The Kaplans developed the theory of recovery of attention, and the theory asserted that the natural environment can restore people's attention and has a significant effect on stress relief (26).

Previous study showed that physiological and physiological indicators of human can feed back quickly; anaerobic exercise can cause changes in systolic blood pressure transiently; heart rate could restore to normal in 5 minutes within climbing competition (27, 28). The human body's psychological perception is also rapidly changing, which can be confirmed by facial expressions changing at any time (29–32). Some experiments have shown that short-term recoveries of 2–10 min are better for health recovery by watching beautiful scenery videos or pictures (33–35). Short-term stay is especially important for students who are rushing to class and back to the dormitory. Hence, the transient effect of the environments on students' physiological and psychological health should not be ignored.

Frequent visits to green spaces by college students can improve their overall mood and reduce perceived stress (36). The objective of this study was to assess the transient recovery effect of different campus spaces on students' physiological and psychological health and contribute to the construction of campus landscapes in the future. We tested participants' physiological and psychological indicators for 5 min since they can reflect the transient recovery effect. We hypothesized that: (i) campus environment has an impact on the transient recovery of students' physiological and psychological health after stress; (ii) blue space, gray space, and green space of campus environment have different recovery effects; and (iii) physiological indicators of systolic blood pressure, diastolic blood pressure, heart rate have a positive correlation with physiological indicators of being away, extent, fascination, and compatibility.

## 2. Materials and methods

### 2.1. Study sites and landscape description

**Figure 1:**
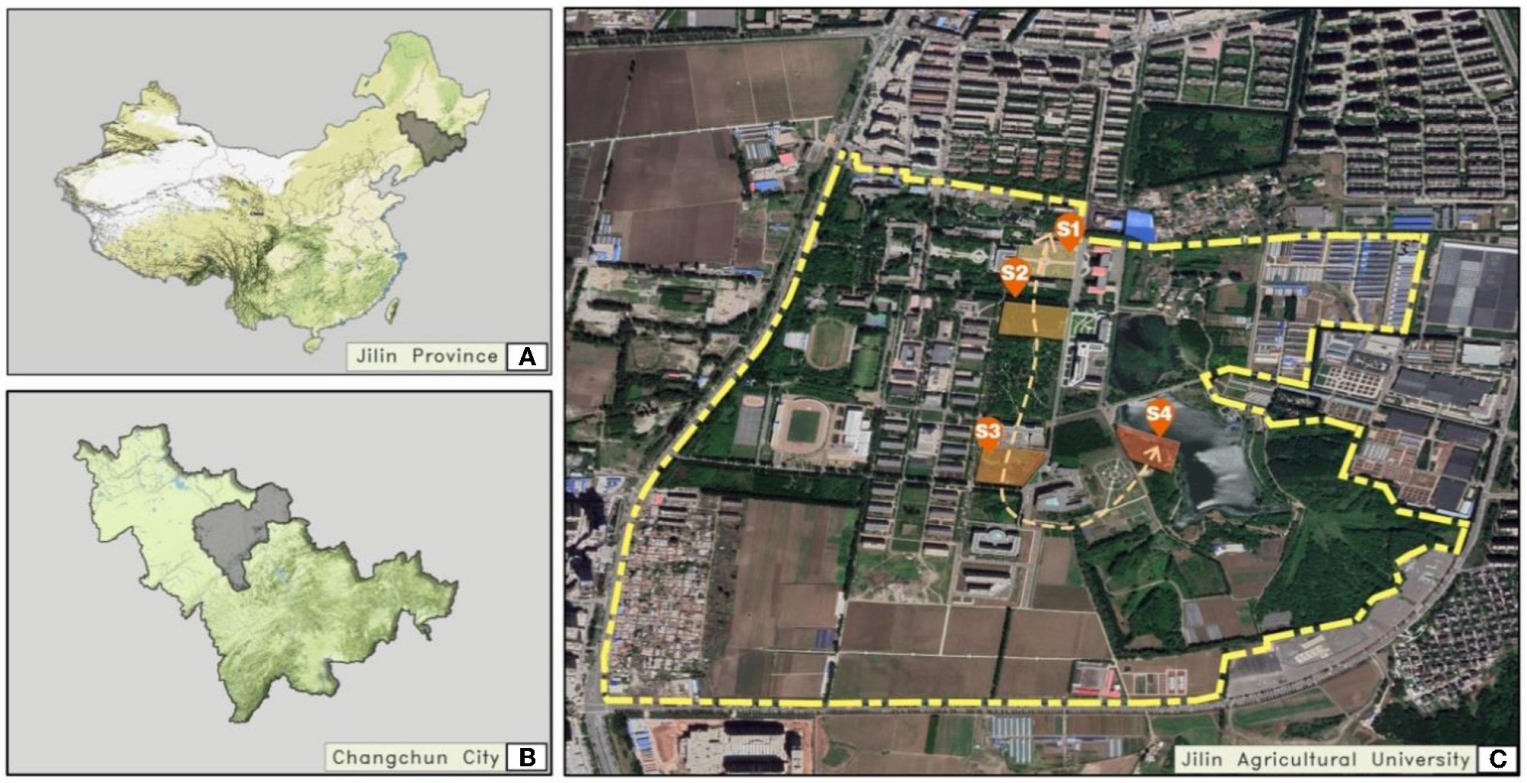
Map of experimental location. **(A)** Jilin Province, China; **(B)** Changchun City, Jilin Province; **(C)** Jilin Agricultural University; (s1–s4) Study Sites.

To assess the effect of different campus spaces on transient recovery of physiological and psychological health, a field experiment was conducted in September 2021 in Changchun, Jilin Province, China. All of the sites that were used in this study are located at Jilin Agricultural University in Changchun. Jilin Agricultural University is one of the key institutions of higher education in Jilin Province and encompasses an area of 1,347 ha, of which 312.66 ha is covered by the campus (Figure 1).

**Figure 2:**
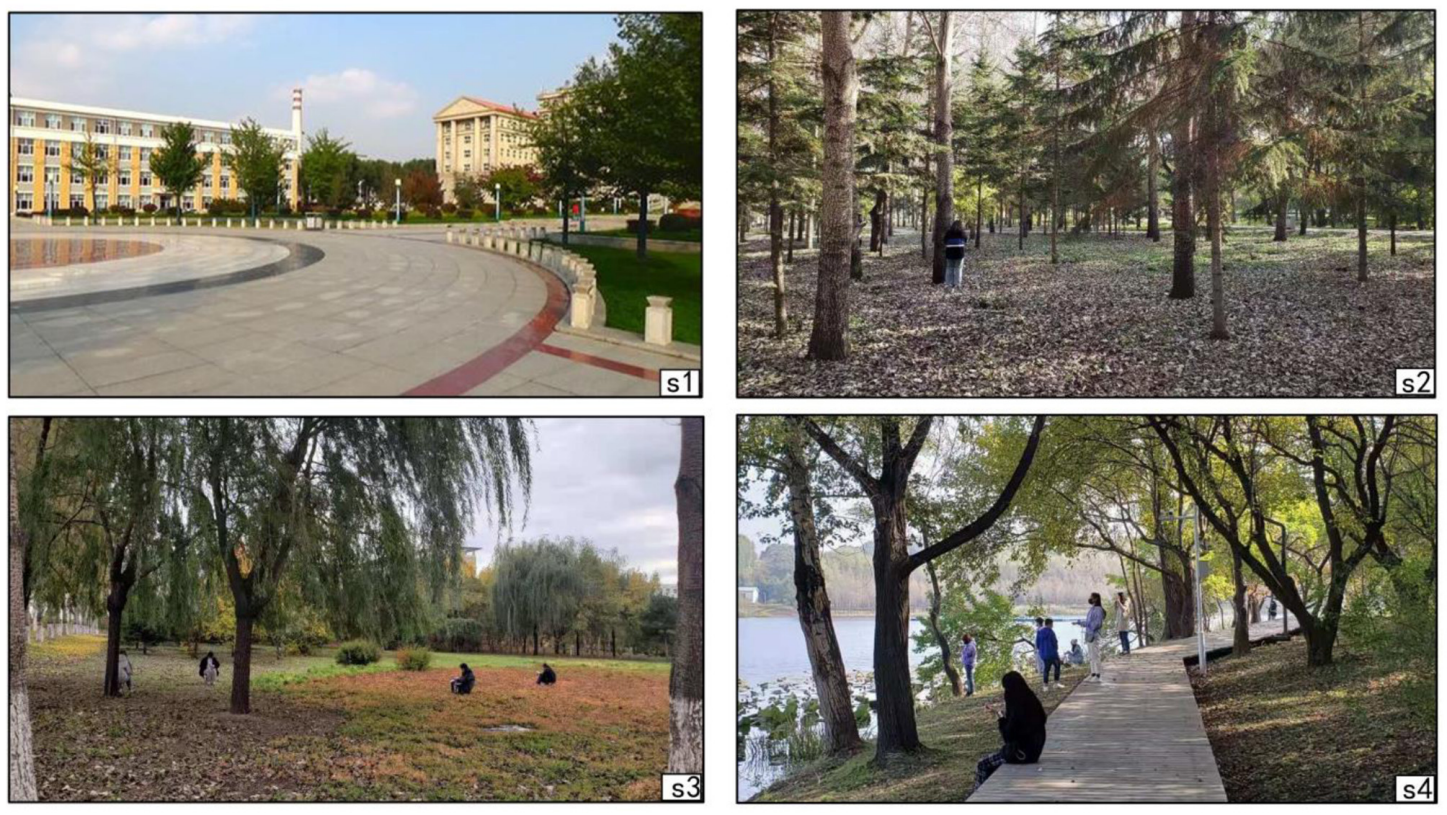
Research locations. **(s1)** Square space; **(s2)** dense forest space; **(s3)** sparse forest space; and **(s4)** waterfront space.

The sampling spaces on the campus of Jilin Agricultural University were selected according to different types of campus landscapes. They are gray spaces, green spaces, and blue spaces. Therefore, sample site 1 (s1) was a square space for students to stay and relax, sample site 2 (s2) was a dense forest space (denseness > 0.70), sample site 3 (s3) was a sparse forest space (denseness < 0.10–0.20, excluding 0.20), and sample site 4 (s4) was a waterfront space, and the control space was a classroom with a high usage rate for daily study (Figure 2).

### 2.2. Study subjects and grouping

The subjects were second-year Landscape Architecture major students at Jilin Agricultural University. Students with heart disease or other medical histories were excluded. Previous studies have shown that environmental design professionals are more sensitive to landscape aesthetic evaluation and have better environmental discrimination than their non-professional counterparts. Therefore, we chose students with a professional background as subjects (37). Prior to the experiment, the objectives of the study and the experimental procedures were explained to the participants, and informed consent was obtained. The subjects were also informed that any strenuous physiological activity, smoking, and alcohol consumption were prohibited. Wearing comfortable clothes and no masks throughout the pre-experimental period and during the experiment was required, and any individual who did not agree or who did not wish to continue participating in the experiment was excluded during the experiment. Sixty subjects were randomly divided into 10 groups of six subjects each. In addition, the subjects' basic information about blood pressure, heart rate, and recent pressure was measured and tallied. According to the data, we found recent stress of the subjects was similar, and the physiological indicators were quite different, but each subject was tested at all the sites, so this individual difference did not affect the results (Table 1).

**Table 1:** Basic information of different subjects in this study (*n* = 60).

**Parameter**	**Age**	**SBP**	**DBP**	**HR**	**Recent Pressure**
Mean	18.48	108.87	72.28	77.75	2.10
SD	0.84	9.11	6.38	10.62	0.60

SD, standard deviation; SBP, systolic blood pressure; DBP, diastolic blood pressure; HR, heart rate.

### 2.3. Experimental design

This study was conducted from 8:30 a.m. to 12:00 a.m. on 25 September 2021. All participants were randomly divided into 10 groups. With the aim to make every participant visit four kinds of green space in the morning, five groups completed the test according to the walking route of square space—dense forest space -sparse forest space—waterfront space, the other groups according to the walking route of waterfront—sparse forest space—dense forest space—square space. It takes about 5 min walking between any two sites in the route. Each group left half an hour apart to avoid interference between different groups in the same campus space. We asked the subjects to walk at a constant speed without running, jumping, or talking. During the preparation phase of each site, the subjects were guided to freely stroll into the vicinity of the sample site (the isolated test environment), where the test procedure was explained. The subjects regained their composure and completed the first part of the questionnaires regarding psychological indicators and the test apparatus was worn. Then the volunteers were assigned to watch a stress video lasting 5 min during which the test apparatus was worn continuously. The guide started to record the physiological indexes after 5 min. After the test, the second part of the questionnaire was completed (Table 2).

**Table 2:** Time schedule of different recovery measures.

**Test process**		**Duration**
Preparation stage	Informed consent, interpretation scale	5 min
	Fill out the scale, baseline blood pressure, and heart rate	1 min
Test stage	Pressure	5 min
	Resume blood pressure and heart rate measurement	5 min
	Fill in the scale to restore the dimensional evaluation	4 min

The overall route of the experiment involved square space (SS), dense forest space (DFS), sparse forest space (SFS), and waterfront space (WS). SS meant the Chongzhi Square; DFS meant the dense forest space within the campus adjacent to the academic buildings; SFS meant the sparse forest space involved the sparse forest space corresponding to the library; WS meant the experimental site selected on the shore of the Blue Lake.

### 2.4. Sample space and methods of measuring physiological and psychological indicators

#### 2.4.1. Physiological indicators

Biofeedback measurements were used to evaluate physiological changes, including blood pressure (diastolic blood pressure and systolic blood pressure) and heart rate (38). A Scian wrist sphygmomanometer was used to collect the physiological parameters from the subjects during the trial (LD-735, Lude Medical Instruments Co., Ltd., Shanghai, China), including diastolic blood pressure, systolic blood pressure, and heart rate. To minimize measurement errors, the data were averaged over three iterations.

#### 2.4.2. Psychological variables

This study used the restorative components scale (RCS) proposed by a 2001 study by Laumann and other scholars as a questionnaire to explore the influence of the landscape environment on subjects' physiological health recovery (39). The RCS is based on the environmental restorative scale (PRS) and remedies some of the noted shortcomings of the PRS. The questionnaire was divided into two main sections: basic information about the subject and the RCS and divided the scale into four themes: being away, extent, fascination, and compatibility (40). Being away refers to distancing from the disturbing negative stimuli in the environment, from people's pursuit of their own goals in life, and from the circumstances, responsibilities, and obligations of daily life (41). Extent means that the environment has a good and broad view and that the place and the surrounding wider environment are organically connected, in harmonious coexistence, and not abrupt. Fascination refers to the natural environment itself having its charm. The effect of restoring could achieve, whatever the person whether put themself on the scene. Compatibility means that the environment is conducive to people's meditation and recovery from mental fatigue. Compatibility requires that the environment, personal preferences, and behaviors required by the environment reach an appropriate and balanced level (42, 43). In addition, there were four to six items for each theme, and the scale was scored on each item by using the five levels of the Likert scale (Table 3).

**Table 3:** Four themes of restorative components scale and items.

**Four themes of restorative components scale**	**Items**
Being away	1. I do something different than I usually do
	2. I am in a different environment than usual
	3. When I am here I feel free from work and routine
	4. When I am here I feel free from other peoples' demand and expectations
	5. I am away from my obligations
	6. I'm free from the demands and expectations of others
Extent	7. The surroundings are coherent
	8. The elements here go together
	9. All the elements constitute a larger whole
	10. The existing elements belong here
Fascination	11. There is plenty to discover here
	12. There are many things here that I find beautiful
	13. There is plenty that I want to linger on here
	14. There are many objects here that attract my attention
	15. I am absorbed in these surroundings
Compatibility	16. The environment gives me the opportunity to do activities that I like
	17. I can handle the kinds of problems that arise here
	18. There is an accordance between what I like to do and these surroundings
	19. I rapidly adapt to this setting
	20. I am capable of meeting the challenge of this setting

Being away includes questions 1–6, extent includes questions 7–10, fascination includes questions 11–15, and compatibility includes questions 16–20.

### 2.5. Data analysis

All data were analyzed by using SPSS 26.0 (IMB SPSS Statistics, Chicago, IL, USA). Continuous data that conformed to a normal distribution were statistically described by their means with standard errors. Abnormal data were statistically described by Whisker–Box plots with medians and quartiles disclosed (44). A Wilcoxon non-parametric test was used for comparing physiological and psychological parameters among different sites. A Kruskal–Wallis (H) non-parametric test was used for the comparison of changes in physiological and psychological indicators before and after pressure (29). Spearman's correlation was used for correlation analysis. Significant statistics were identified by *P*-values of < 0.05.

## 3. Results

### 3.1. Effect of different sites on physiological and psychological indicators

**Figure 3:**
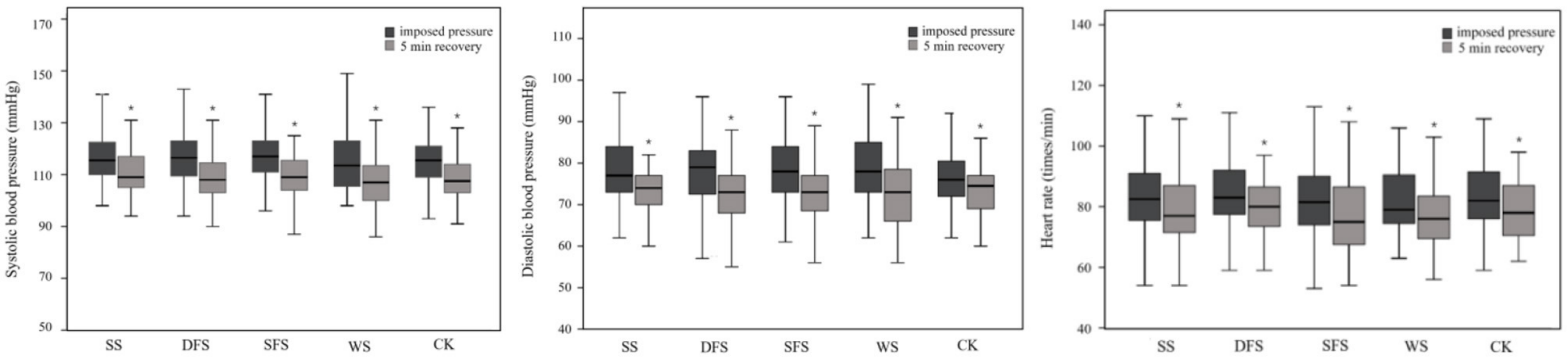
Comparison of different physiological indicators. SS, square space; DFS, dense forest space; SFS, sparse forest space; WS, waterfront space; CK, control check group; SBP, systolic blood pressure; DBP, diastolic blood pressure; HR, heart rate. *Represents statistical significance compared with that after compression.

The physiological and psychological indicators were abnormal, so a Whisker–Box was adopted with medians and quartiles to describe the data. The blood pressure and heart rate measurements of the volunteers in the five sites changed significantly after 5 min of recovery (*P* < 0.05), and the median values of systolic blood pressure, diastolic blood pressure, and heart rate all decreased. The test showed that the systolic blood pressure, diastolic blood pressure, and heart rate after 5 min of recovery were significantly different from the systolic blood pressure, diastolic blood pressure, and heart rate after pressure, and the results showed that all sites had positive effects on the physiological indicators of the volunteers (Figure 3).

**Figure 4:**
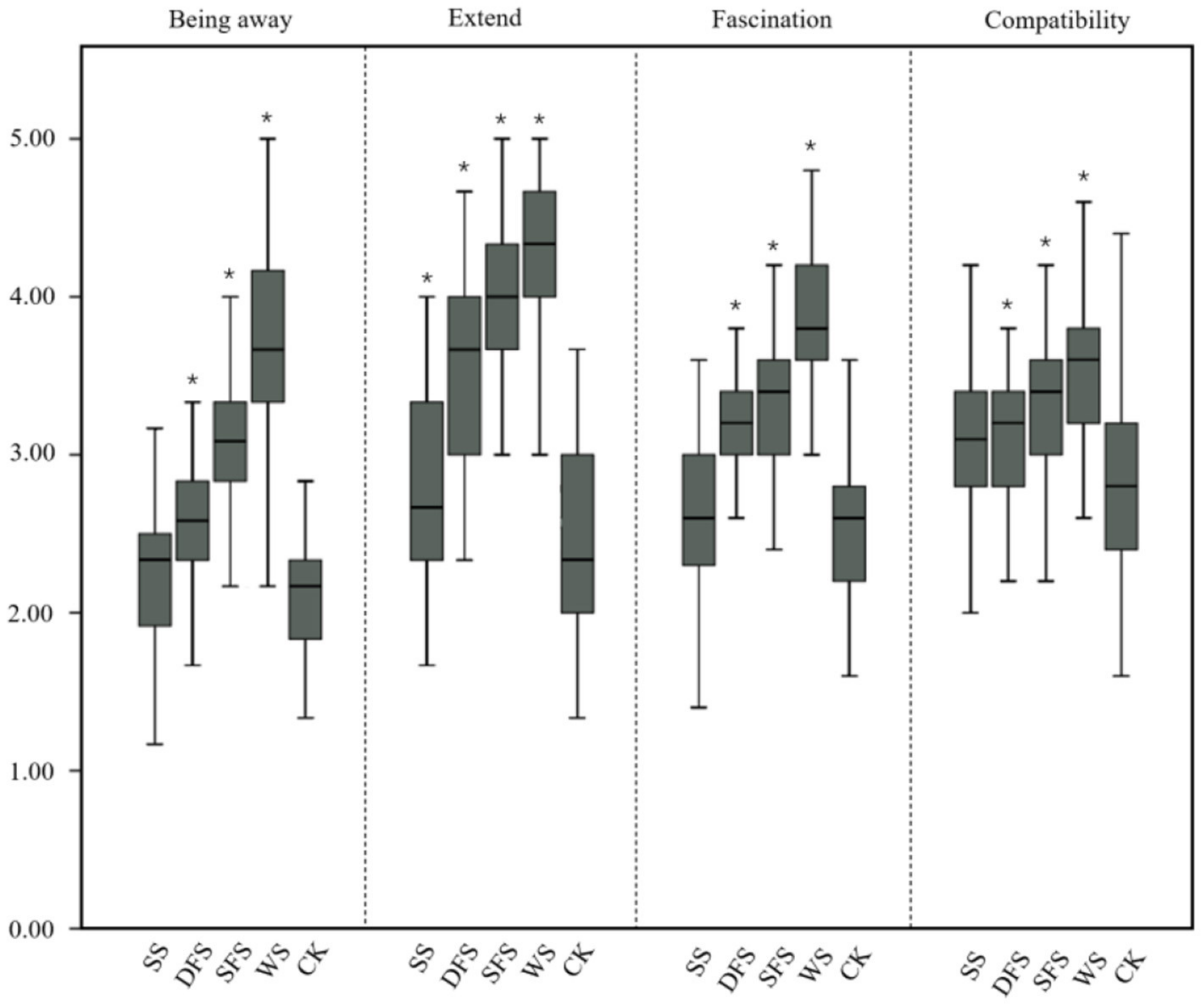
Comparison of different psychological indicators. SS, square space; DFS, dense forest space; SFS, sparse forest, and grass space; WS, waterfront space; CK, control checkgroup. *Represents statistical significance of means compared after compression.

In terms of psychological recovery, the RCS scores of the five sites were all positive after 5 min of recovery. Referring to the RCS scores of the five sites after pressure, all sites had restorative effects on psychological health, but the recovery degree was different. The recovery effects are in increasing order as follows: waterfront space > sparse forest space > dense forest space > control check group. The waterfront space, sparse forest space, and dense forest space had significant differences from the square space and the control check group in the four dimensions of psychological recovery (Figure 4).

The results showed that different campus spaces were beneficial to the transient recovery of students' physiological and psychological health, but there was no significant difference. However, the waterfront space, sparse forest space, and dense forest space had significant differences in the transient recovery effect of students' psychological health.

### 3.2. Comparison between physiological and psychological recovery amounts

**Figure 5:**
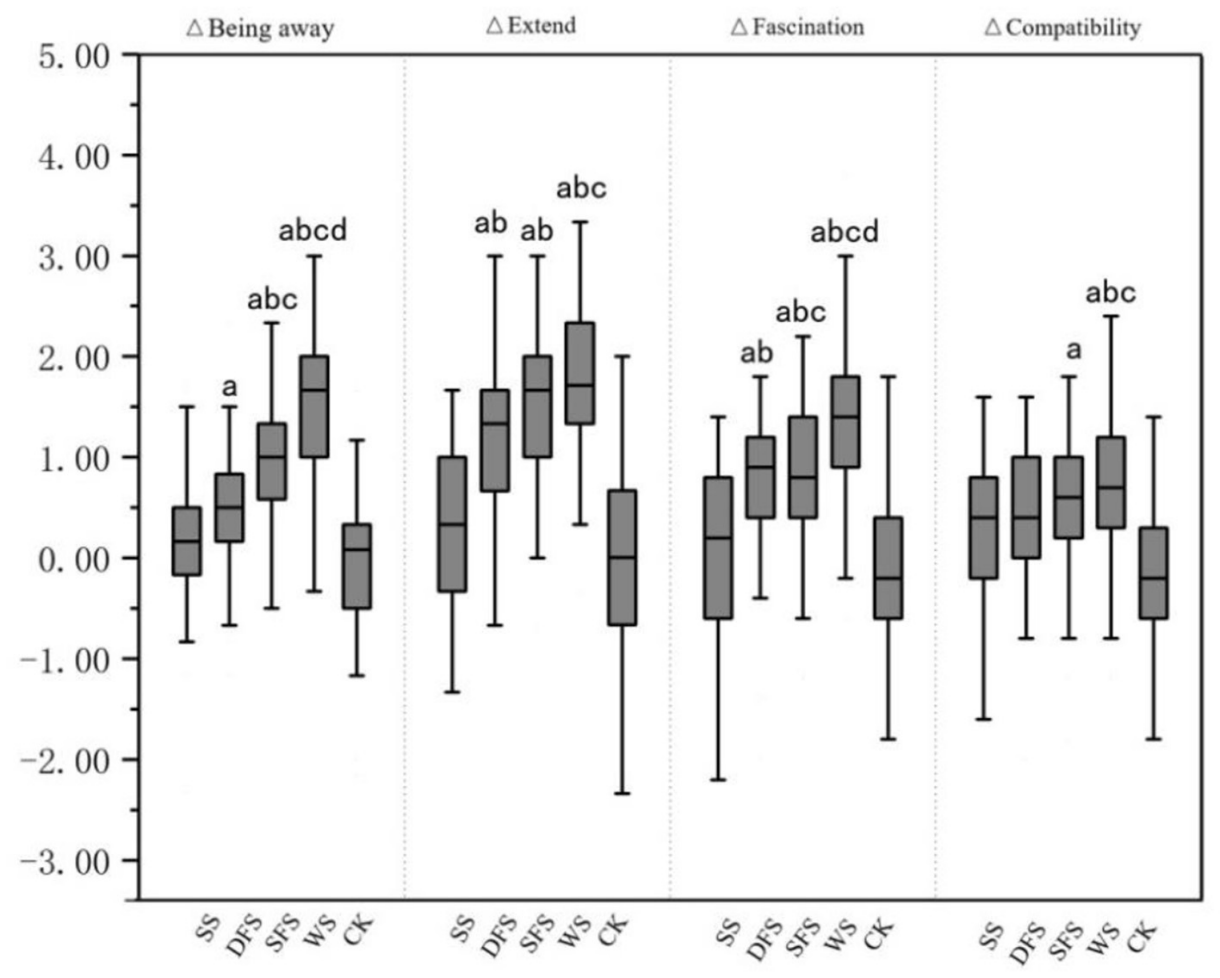
Comparison of psychological recovery differences of the five sites. SS, Square space; DFS, dense forest space; SFS, sparse forest space; WS, waterfront space; CK, control check group. a. Compared with control, *P* < 0.05; b. Compared with SS, *P* < 0.05; c. Compared with DFS, *P* < 0.05; d. Compared with SFS, *P* < 0.05. No marks mean no significant difference.

Systolic blood pressure, diastolic blood pressure, and heart rate recovery amounts (recovery amounts = the fifth-min recovery index – imposed pressure index) of the five sites were all negative, indicating that systolic blood pressure, diastolic blood pressure, and heart rate indicators decreased after the 5 min recovery. However, there was no statistical difference in systolic blood pressure, diastolic blood pressure, and heart rate recovery amounts across the five sites, indicating that different sites had the same ability to restore volunteers' physiological health transitorily. In terms of the recovery of psychological indicators, the five sites all had positive scores, proving that psychological health can be improved just by getting rid of your stressors (Figure 4). However, the psychological recovery amounts (recovery amounts = the fifth-min recovery index – imposed pressure index) of being away, extend, fascination, and compatibility of the other four sites were all positive except for the control check group, indicating that the four sites had a recovery effect on psychological health transitorily. In addition, there were significant statistical differences in four psychological perception dimensions about four sites (Table 4). By further pairwise comparison, in terms of being away: water space > sparse forest space > dense forest space, in terms of extent: water space = sparse forest space > dense forest space, in terms of fascination: water space > sparse forest space = dense forest space, in terms of compatibility recovery: water space > sparse forest space = dense forest space (Figure 5). A comparison of the two methods showed that the water space was the most conducive to transient psychological recovery of all the campus spaces.

**Table 4:** Physiological and psychological recovery amounts of the five sites.

**Site**	**ΔSBP**	**ΔDBP**	**ΔHR**	**Δ Being away**	**Δ Extent**	**ΔFascination**	**ΔCompatibility**
SS	−7.00 (−12.00, −1.00)	−4.50 (−9.75, −1.00)	−4.00 (−10.00, 0.75)	0.17 (−0.29, 0.50)	0.33 (−0.25, 0.92)	0.00 (−0.55, 0.60)	0.20 (−0.20, 0.75)
DFS	−7.00 (−11.00, −4.00)	−5.00 (−9.00, 0.00)	−4.00 (−7.00, 1.00)	0.42 (−0.13, 0.83)	1.00 (0.66, 1.67)	0.60 (0.20, 1.15)	0.20 (−0.20, 0.60)
SFS	−7.50 (−14.75, −3.00)	−4.00 (−11.00, −1.00)	−5.00 (−8.00, 0.00)	0.84 (0.33, 1.46)	1.67 (1.33, 2.00)	1.00 (0.25, 1.35)	0.40 (−0.15, 0.80)
WS	−6.50 (−13.75, −2.00)	−6.00 (−11.00, 0.75)	−5.00 (−10.00,−3.00)	1.58 (1.00, 2.12)	2.00 (1.33, 2.34)	1.30 (0.80, 1.80)	0.60 (0.20, 1.00)
CK	−5.00 (−12.00, −1.00)	−3.50 (−8.00, 1.75)	−5.50 (−10.75, 0.75)	0.08 (−0.50, 0.33)	0.00 (−0.66, 0.67)	−0.20 (−0.60, 0.40)	−0.20 (−0.60, 0.35)
P	0.593	0.606	0.796	0.000	0.000	0.000	0.000

SS, square space; DFS, dense forest space; SFS, sparse forest space; WS, waterfront space; CK, control check group. SBP, systolic blood pressure; DBP, diastolic blood pressure; HR, heart rate; Δ = 5 min recovery index—imposed pressure index.

### 3.3. Relationship between the physiological and psychological indicators

The results of the correlation analysis showed that there was a certain degree of correlation between psychological and physiological indicators after 5 min of recovery. There was a significant positive correlation between fascination and diastolic blood pressure in square space, dense forest space, and waterfront space. That is, diastolic blood pressure increased with the increase in fascination scores, and high fascination slowed down the recovery of blood pressure. There was a significant positive correlation between compatibility and heart rate in the square space and the dense forest space, indicating that the higher the score of compatibility, the higher the heart rate, and the lower the recovery degree of the physiological index heart rate. Compatibility also had a positive correlation with systolic blood pressure and diastolic blood pressure in the sparse forest space, indicating that the higher the score of compatibility, the lower the recovery degree of the systolic blood pressure and diastolic blood pressure. Last but not least, no regular changes in the correlation between the being away, extent, and physiological indexes (Table 5).

**Table 5:** Correlation between physiological indicators and psychological indicators.

**Comparison index**		**Recovery being away**	**Recovery extent**	**Recovery fascination**	**Recovery compatibility**
SS	Recovery SBP	0.349	0.098	0.476	0.536
	Recovery DBP	0.331	0.756	0.006^**^	0.742
	Recovery HR	0.177	0.287	0.376	0.022^*^
DFS	Recovery SBP	0.677	0.441	0.121	0.774
	Recovery DBP	0.930	0.580	0.024^*^	0.129
	Recovery HR	0.431	0.044^*^	0.111	0.035^*^
SFS	Recovery SBP	0.101	0.283	0.071	0.022^*^
	Recovery DBP	0.252	0.042^*^	0.757	0.023^*^
	Recovery HR	0.102	0.377	0.910	0.934
WS	Recovery SBP	0.957	0.275	0.075	0.330
	Recovery DBP	0.962	0.398	0.002^**^	0.069
	Recovery HR	0.827	0.632	0.898	0.386
CK	Recovery SBP	0.793	0.052	0.568	0.618
	Recovery DBP	0.656	0.217	0.598	0.818
	Recovery HR	0.757	0.976	0.009^**^	0.987

SS, square space; DFS, dense forest space; SFS, sparse forest and grass space; WS, waterfront space; CK, control group. SBP, Systolic blood pressure; DBP, diastolic blood pressure; HR, heart rate. ^*^Correlation is significant at the 0.05 level. ^**^Correlation is significant at the 0.01 level.

The correlation analysis results of different recovery amounts within 5 min of all sites were shown that there was a weak correlation between recovery amounts. The only thing we should pay attention to is the significant positive correlation between the recovery amount of diastolic blood pressure and the recovery amount of being away in the sparse forest space, that is to say, the greater the change in being away, the greater change in diastolic blood pressure. In the control check group, there was a significant positive correlation between heart rate recovery and recovery amounts due to fascination, indicating that the greater the change in fascination, the greater the change in heart rate (Table 6).

**Table 6:** Correlation between the recovery amounts of the physiological indicators and the psychological indicators.

**Comparison index**		**Δ Being away**	**Δ Extend**	**Δ Fascination**	**Δ Compatibility**
SS	ΔSBP	0.642	0.184	0.141	0.847
	ΔDBP	0.107	0.146	0.063	0.073
	ΔHR	0.257	0.110	0.633	0.580
DFS	ΔSBP	0.145	0.073	0.051	0.445
	ΔDBP	0.925	0.433	0.749	0.909
	ΔHR	0.369	0.165	0.143	0.468
SFS	ΔSBP	0.059	0.750	0.358	0.793
	ΔDBP	0.023^*^	0.334	0.543	0.719
	ΔHR	0.289	0.628	0.636	0.405
WS	ΔSBP	0.584	0.847	0.709	0.703
	ΔDBP	0.951	0.068	0.747	0.761
	ΔHR	0.073	0.797	0.529	0.035
CK	ΔSBP	0.164	0.913	0.352	0.903
	ΔDBP	0.901	0.562	0.779	0.736
	ΔHR	0.950	0.237	0.041^*^	0.839

ΔSBP, systolic blood pressure recovery amounts; ΔDBP, diastolic blood pressure recovery amounts; ΔHR, heart rate recovery amounts. ΔBeing away, being away recovery amounts; ΔExtend, extend recovery amounts; ΔFascination, fascination recovery amounts; ΔCompatibility, compatibility recovery amounts. ^*^Correlation is significant at the 0.05 level.

The results showed that the transient recovery of blood pressure was closely related to a fascination with the environment, and heart rate recovery was closely related to the compatibility of the environment. In terms of recovery amounts, the sense of escape helps to restore blood pressure.

## 4. Discussion

### 4.1. Restoration of physiological health

Repeat visits or prolonged visits to the natural environment have positive effects on physiological and psychological health and the differential effects of natural and urban environments can appear within 4 min in physiology (45–47), which is consistent with the conclusion of this study. In our study, subjects' physiological indexes in five sites on campus were recovered within 5 min (Figure 3), but there was no significant difference in the recovery amounts of different campus space types (Table 4). This indicates that physiological indicators can quickly feedback within 5 min, and the differential response of physiological indicators cannot be caught after 5 min. This may be due to the differential performance of physiological indicators ending after 5 min. Previous studies have also shown that visiting campus green landscapes can reduce college students' blood pressure and significantly increase their positive emotions, and the restorative effects of the environment are due to perceptions about vegetation, water, and chairs (3, 48). Perhaps this can be explained by the preference for landscape elements that are not immediately apparent in our study. Finally, there could be another reason that the sensitivity of the test equipment was not good. In future experiments, we could choose more sensitive equipment, such as skin sensors, to measure transient changes in physiological indicators, or extend the test time to 10 min.

### 4.2. Restoration of psychological health

Nature is closely related to happiness, and a stronger connection to nature can lead to greater happiness. Nature affects emotions more slowly and over a longer period of time than it affects physiology (46, 49). Some studies have suggested that green spaces have a good restorative effect, and blue spaces also have a good restorative potential (50). The waterfront space, through questionnaires, has been shown the optimal perceived attention restoration effects, followed by vegetation spaces, courtyard spaces, and square spaces (36). These are consistent with our findings, and the difference in psychological recovery was still seen after 5 min. The results in this study showed that the square space and control check group had a weak effect on the transient recovery of psychological health after pressure, the psychological indicators of the other three sites recovered significantly 5 min after pressure. It means that green plants and blue water have positive effects on psychological recovery (Figure 4). The water space on campus was the best choice for transient psychological recovery after pressure, and the sparse forest space was better for transient psychological recovery after stress than the dense forest space (Figure 5). This is consistent with the opinion that garden pieces and the environment, plant species, waterscape state, and boundary clarity were identified as significant landscape elements with perception-restorative effects (51). Some studies also have shown that the impact of blue-green space on mental restoration varies with the seasons, which of course is closely related to meteorological factors (52). However, our study was conducted in late summer, so the results can only illustrate the restorative effects of different campus spaces on the psychological health of students during the summer.

### 4.3. Relationship between changes in physiological and psychological variables

Exposure to the natural environment has effects on both physical and psychological health. The effects will appear quickly and complement each other, e.g., high psychological stress can lead to changes in blood pressure, and extraversion is associated with blood pressure in adolescents (46, 53, 54). This is consistent with previous studies. Our study showed that physiological and psychological recovery interacted within 5 min. There was a significant positive correlation between fascination and diastolic blood pressure in space square, dense forest space, and waterfront space after 5 min of recovery, meaning that interesting features of the environment slowed the transient recovery effects of diastolic blood pressure. The results also show that there was a significant positive correlation between compatibility and heart rate in the square space and the dense forest space, meaning that a comfortable environment for relaxation and socializing leads to slower transient heart rate recovery. This may be due to the sympathetic arousal caused by the interesting and comfortable landscape or the stress video eliciting unconscious attention or fascination, resulting in a stress response, so the recovery of diastolic blood pressure and heart rate was slower. In terms of 5 min recovery amounts, diastolic blood pressure and being away were correlated in the sparse forest space. Thus, the restorative effect of the sparse forest space was more closely related to its sense of being away.

We also found no correlation between the sense of being away and recovery of physiological measures after 5 min of recovery. The reason for this may be the result of cognition; people already subconsciously distinguished the natural environment from the urban environment and imagining the forest and water environment had already triggered relaxed, good emotions. In terms of 5 min of recovery, although there was a correlation between the sense of being away and blood pressure in the sparse forest space, the overall correlation between psychological and physiological indicators was weak. The weak correlation between physical and psychological indexes may be due to the fact that explicit positive or negative emotional responses to natural stimuli can occur in 400 milliseconds or less, and that nature may have beneficial psychological effects based on subjective judgments which provide limited assessments of stress recovery. Therefore, different measurement modes lead to a weak correlation between objective recovery on physiological and psychological aspects (47). The results also suggest that theories that emphasize attention or attraction may not be enough to fully explain the restorative properties of nature and that we could also focus on differences in stressors or whether attractiveness or attraction can trigger stress again.

## 5. Limitations

In this study, we obtained results on the impact of different spaces on campus on the transient recovery of young students' stress parameters. The environmental factors and the volunteers' physiological and psychological indicators, as well as their correlations, were quantitatively analyzed. However, this study also had several shortcomings. First, due to time constraints, this study was only conducted in late summer, and future studies need to examine the transient recovery capacity of campus spaces for stress in different seasons and time periods. Second, the testing of environmental factors could also include ecological factors, which would help to obtain a more accurate correlation analysis. Finally, the tests of physiological indicators could also include more sophisticated instruments, such as the use of eye movement instruments and skin sensors.

## 6. Conclusion

The physiological and psychological data from this campus trial provide an important scientific basis for the capacity of different campus environments for transient recovery. In terms of positive changes in physiological indicators from stress to 5 min recovery, systolic blood pressure, diastolic blood pressure, and heart rate decreased significantly in the short term in the five sites, but there was no difference in recovery amounts of the five sites. For the psychological recovery effects, the psychological indicators in the dense forest space, sparse forest space, and waterfront space had positive changes, and there were significant differences in the recovery amounts in increasing order as water space > sparse forest space>dense forest space. It is suggested that the water space was beneficial for college students to quickly relieve their mood and free themselves from pressure quickly, and the transient recovery effect of the sparse forest space with low canopy density was better than the dense forest space with high canopy density. Therefore, attention should be paid to the following aspects in the construction of campus waterfront landscapes. First, the function of the waterfront landscape should be optimized, scenic spots should be arranged according to a certain distance, and the viewing space for staying and stopping should be appropriately set up to meet the requirements of students' daily leisure space. Second, the richness of waterfront space facilities and the diversity of use functions should be enhanced. A reasonable planning mode should be set on pedestrian area road which should be set in beautiful scenery,closer by landscape node. Finally, in terms of plant collocation, the construction of the plant landscape system should be enriched. The combination of native tree species and exotic domesticated tree species should be selected to create a visual landscape effect with considerable scenery in all seasons and a rich visual landscape effect while ensuring a certain openness and security.

From the correlation of physiological and psychological indicators after 5 min of recovery, the fascination score was most strongly correlated with diastolic blood pressure and compatibility had an impact on systolic blood pressure, diastolic blood pressure, and heart rate. Thus, whether the environment is attractive and conducive to activities has a great impact on the physiological index recovery transiently, although the effect is to hinder recovery. For the correlation between physiological and psychological recovery amounts within 5 min, only the difference in being away and the difference in diastolic blood pressure is correlated with sparse forest space, that is, the greater the difference in being away, the greater the difference in diastolic blood pressure. Thus, escaping from daily chores is the most important factor in the transient recovery process for the physiological indicators. However, the overall correlation between physiological and psychological indicators in transient recovery effect was not strong, that may be due to physiological changes being so rapid for them to detect; the psychological evaluation of this experiment being a subjective evaluation with certain errors; preferences for different landscape spaces not being quickly reflected in the physiological indexes. Explaining the restorative effects of nature on humans' physiological and psychological health may require more theoretical support. Thus, the campus environment creation for short-term stays of space in the future, waterfront space should be emphasized, following sparse forest space and final dense forest space; because these spaces have a transient recovery effect on the students' physiological and physiological health. Furthermore, too many artificial facilities in these landscape spaces should be avoided, because charming scenery and activities will hinder physiological and psychological health recovery. Our conclusions may also be instructive for the construction of some sites in cities that are suitable for short-term natural exposure, and give urbanites a release from life troubles.

## Data availability statement

The raw data supporting the conclusions of this article will be made available by the authors, without undue reservation.

## Ethics statement

Ethical review and approval was not required for the study on human participants in accordance with the local legislation and institutional requirements. The patients/participants provided their written informed consent to participate in this study.

## Author contributions

WN and XS: conceptualization. WN: methodology, writing—original draft preparation, visualization, and funding acquisition. JY: formal analysis and resources. XS: investigation and writing—review and editing. QC: supervision. All authors have read and agreed to the published version of the manuscript. All authors contributed to the article and approved the submitted version.
